# The Role of Multi-modality Imaging in the Diagnosis of Cardiac Amyloidosis: A Focused Update

**DOI:** 10.3389/fcvm.2020.590557

**Published:** 2020-10-30

**Authors:** Shaun Khanna, Ivy Wen, Aditya Bhat, Henry H. L. Chen, Gary C. H. Gan, Faraz Pathan, Timothy C. Tan

**Affiliations:** ^1^Department of Cardiology, Blacktown Hospital, Sydney, NSW, Australia; ^2^School of Medical Sciences, University of New South Wales, Sydney, NSW, Australia; ^3^Department of Cardiovascular Imaging, Nepean Clinical School, University of Sydney, Sydney, NSW, Australia

**Keywords:** cardiac amyloidosis, global longitudinal strain, speckle tracking echocardiography, cardiac MRI, CT cardiac scan, pet scan, DPD scintigraphy

## Abstract

Cardiac amyloidosis (CA) is a unique disease entity involving an infiltrative process, typically resulting in a restrictive cardiomyopathy with diastolic heart failure that ultimately progresses to systolic heart failure. The two most common subtypes are light-chain and transthyretin amyloidosis. Early diagnosis of this disease entity, especially light-chain CA subtype, is crucial, as it portends a poorer prognosis. This review focuses on the clinical utility of the various imaging modalities in the diagnosis and differentiation of CA subtypes. This review also aims to highlight the key advances in each of the imaging modalities in the diagnosis and prognostication of CA.

## Introduction

Amyloidosis is a heterogeneous disease process of systemic unrestrained deposition of insoluble amyloid protein in the extra-cellular space of multiple tissues (1). The two most common subtypes described are transthyretin (TTR) and light chain (AL) amyloidosis (1–3). TTR amyloidosis encompasses both familial (mutant) TTR and wild-type TTR, whereas AL amyloidosis is usually associated with an underlying plasma cell dyscrasia (1). The types of cardiac amyloidosis (CA) differ based on the type of misfolded amyloid fibril that is deposited within tissues, ultimately impacting on overall clinical progression, prognosis and treatment (2). AL amyloidosis usually involves abnormal light chains as the amyloidogenic precursor whereas TTR amyloidosis involves an abnormal production of the amino acid transthyretin.

AL amyloidosis has a poorer prognosis when compared to TTR amyloidosis, hence treatment frequently involves chemotherapy and organ transplantation whereas TTR amyloidosis involves non-steroid anti-inflammatory medications and targeted therapies (3). CA occurs when misfolded protein is deposited within the myocytes, a phenomenon which generally heralds a poorer prognosis in this population (4). This infiltrative process frequently results in a restrictive cardiomyopathy, prevalent in approximately half of patients affected with the disease (4, 5).

Endo-myocardial biopsy with histopathological diagnosis is the current gold standard diagnostic method, which may not be feasible in certain cases or routinely available at all centers (4). There is an emerging role for non-invasive diagnostic imaging modalities in the diagnosis of CA, with mounting evidence supporting the utility and value of a range of imaging modalities such as echocardiography (TTE), cardiac magnetic resonance imaging (CMR), bone tracer cardiac scintigraphy (Tc99-PYP scan), cardiac computed tomography (CCT), and positron emission tomography (PET-CT) (6). See Table 1 (7–40). There are several gross morphological changes seen in CA, which are typically consistent across many of the imaging modalities and reflective of underlying pathophysiology. Extracellular amyloid infiltration characteristically leads to thickening of the interstitium of the atrial and ventricular myocardium, with relative sparing of the apex (41). The underlying cause of apical sparing has been hypothesized to be due to less amyloid deposition at the apex, and higher myocyte apoptosis in the basal segments (42). Less frequently, amyloidosis can deposit around coronary arteries, conduction pathways and the pericardium (43). CA therefore has a proclivity to the myocardium, resulting in bi-ventricular wall thickening, with resultant loss of ventricular elasticity. Early disease manifestations include impaired relaxation with preserved myocardial systolic function, progressing to systolic failure with incessant deposition (44). Nonetheless, arriving at the diagnosis can frequently be challenging particularly in the absence of systemic disease (5, 31). The biggest challenge relates to differentiating CA in the early stages of disease process from other common differential diagnoses, namely hypertrophic cardiomyopathy, aortic stenosis, ischemic cardiomyopathies, Fabry's disease and cardiac remodeling from other secondary processes. Therefore, the final diagnosis of CA may require the integration of data from a number of imaging modalities. Moreover, the various imaging modalities have varied sensitivities and specificities for the detection of the different types of amyloidosis secondary to the underlying pathophysiological mechanism of disease, e.g., CMR is more established for the diagnostic workup in AL amyloidosis due to the greater interstitial expansion in cardiac tissue (45), as compared to Tc99-PYP cardiac scan which has a greater role in the diagnosis of TTR amyloidosis. Bone imaging agents such as Tc99-PYP scanning has a higher specificity and sensitivity for TTR amyloidosis as they are easily taken up by a unique calcium mediated mechanism (46). The exact mechanism is largely unknown but is hypothesized to be due to binding of calcium in TTR amyloidosis fibrils to the phosphate domains in injected tracers (47).

**Table 1:** Summary of multi-modality imaging findings in cardiac amyloidosis.

**Study**	**Year**	**Study Design**	**Patient (n)**	**Imaging modality**	**Findings**
Salman et al. (7)	2017	Retrospective	26 Unspecified subtype	TTE	CA patients had increased left atrial area (23.7 ± 7.5 cm^2^ vs. 18.5 ± 4.8 cm^2^, *p* = 0.04) greater interventricular wall thickness (14.4 ± 2.6 mm vs. 9.3 ± 1.3 mm, *p* < 0.001), lower e′ (0.06 ± 0.02 m/s vs. 0.09 ± 0.02 m/s, *p* < 0.001) when compared to controls
Boldrini et al. (8)	2013	Retrospective	198	TTE	h-TTR patients have a higher indexed LV mass when compared to AL-CA patients (213 ± 59 vs. 161 ± 51, *p* < 0.001)
Pagarouelias et al. (9)	2017	Prospective	100 AL 26 h-TTR 5 wt-TTR 9 HCM 40 Hypertension 20	STE	EF:GLS ratio is effective in discriminating CA (AUC, 0.95; 95% CI: 0.89–0.98; *p* < 0.01)
Barros Gomes et al. (10)	2017	Prospective	150	STE	GLS ≥ −14.81 independently predicts all-cause mortality in AL-CA [hazard ratio: 2.68; 95% CI: 1.07–7.13; *p* = 0.03]
Pun et al. (11)	2018	Retrospective	82	STE	Predictors of survival in AL-CA include IVS diameter (HR: 1.29, 95% CI: 1.12–1.49, *p* = 0.0003) E/A ratio (HR: 2.70, 95% CI: 1.81–4.02, *p* < 0.0001) Lateral E/E' ratio (HR: 2.43, 95% CI: 1.45–4.08, *p* < 0.0008)
Senapati et al. (12)	2016	Retrospective	97 Unspecified subtype	STE	High relative regional strain ratio is an adverse prognostic factor in CA (*p* = 0.018) and independently predicts 5-year outcomes [HR 2.45 (1.36–4.40), *p* = 0.003].
Clemmensen et al. (13)	2020	Retrospective	155 Unspecified subtype	Myocardial Work	LV pressure-strain-derived myocardial work was significantly reduced in cardiac amyloid, compared with healthy control (*p* < 0.0001)
Clemmensen et al. (14)	2018	Prospective	35 AL CA 10 h-TTR 5 wt-TTR 10 Control 10	Myocardial Work	Myocardial work efficiency was significantly reduced in cardiac amyloid, compared with healthy control (13 ± 5% vs. 22 ± 5%; *p* < 0.0001)
Fontana et al. (15)	2015	Prospective	250	CMR	Transmural LGE more prevalent in ATTR (subtype unspecified) than AL (63% vs. 27%, *p* < 0.0001) Subendocardial LGE more prevalent in AL than ATTR (39% vs. 24% < 0.05) Transmural LGE is a significant predictor of mortality (HR, 5.4; 95% CI: 2.1–13.7; *p* < 0.0001) Survival at 24 months is decreased in patients with transmural enhancement, compared to subendocardial enhancement, and compared to no enhancement (60% vs. 80% vs. 90%)
Dungu et al. (16)	2014	Retrospective	97 wt-TTR 28 h-TTR 23 AL 46	CMR	Left ventricular mass increased in TTR compared with AL-CA (202–267 g vs. 137–191 g, *p* < 0.0001) Transmural LGE demonstrated in 90% of TTR compared with 37% of AL-CA (*p* < 0.0001)
Hosch et al. (17)	2007	Retrospective	19 Unspecified subtype	CMR	Patients with CA have increased native T1 relaxation time compared with control [Mean ± SD (95% CI:) 1,340 ± 81 (1,303–1,376) ms vs. 1,146 ± 71 (1,096–1,196) ms, *p* < 0.0001]
Fontana et al. (18)	2014	Prospective	172	CMR	T1 relaxation time is greater in AL compared with ATTR (subtype unspecified) (AL 1,130 ± 68 ms vs. ATTR 1,097 ± 43 ms, *p* = 0.01). T1 tracked cardiac amyloid burden determined by DPD scintigraphy (*p* < 0.0001)
Karamitsos et al. (19)	2014	Prospective	53	CMR	Elevated T1 relaxation time correlates with decreasing LVEF (*r* = −0.57, *p* < 0.001), increasing LVMI (*r* = 0.58, *p* < 0.001), increasing E/E' ratio (*r* = 0.45, *p* = 0.001), and shortened E deceleration time (*r* = −0.44, *p* = 0.002) in patients with AL-CA
Ridouani et al. (20)	2018	Prospective	44 AL 24 wt-TTR 11 h-TTR 9	CMR	Myocardial T2 relaxation times increased in AL-CA compared with ATTR (63.2 ± 4.7 ms vs. 56.2 ± 3.1 ms, *p* < 0.0001). ECV was the best predictor of outcome (HR 1.66 per 0.1 increase in ECV (1.24–2.22); *p* = 0.0006
Kotecha et al. (21)	2018	Prospective	286 Unspecified subtype	CMR	Myocardial T2 was highest in untreated AL patients (untreated AL amyloidosis 56.6 ± 5.1 ms; treated AL amyloidosis 53.6 ± 3.9 ms; ATTR amyloidosis 54.2 ± 4.1 ms; each *p* < 0.01 compared with control subjects: 48.9 ± 2.0 ms). T2 predictive of mortality in AL following correction for ECV and pro-BNP (HR 1.32; 95% CI: 1.05–1.67)
Barison et al. (22)	2014	Prospective	36 Unspecified subtype	CMR	Cardiac ECV was higher in patients with amyloid in comparison to controls (0.43 ± 0.12 vs. 25 ± 0.04, *P* < 0.05). Cardiac ECV in patients without late gadolinium enhancement was significantly higher than controls (0.35 ± 0.10 vs. 25 ± 0.04, *p* < 0.05). Myocardial ECV > 0.316 has a sensitivity of 79% and specificity of 97% for discriminating amyloid patients from controls (AUC 0.884).
Banypersad et al. (23)	2012	Prospective	60	CMR	ECV (determined with gadolinium enhanced T1 mapping) is elevated more significantly in AL-CA than in controls (0.40 vs. 0.25, *p* < 0.001). ECV was correlated with cardiac parameters by echocardiography (e.g., Tissue Doppler Imaging [TDI] S-wave *R* = 0.52, *P* < 0.001) and conventional cardiovascular magnetic resonance (e.g., indexed left ventricular mass *R* = 0.56, *P* < 0.001).
Banypersad et al. (24)	2015	Prospective	100	CMR	Mean ECVi was raised in AL-CA (0.44 ± 0.12) as was ECVb (mean 0.44 ± 0.12) compared with healthy volunteers (0.25 ± 0.02), *P* < 0.001 Native pre-contrast T1 was raised in AL-CA (mean 1,080 ± 87 ms vs. 954 ± 34 ms, *P* < 0.001). ECV is an independent predictor for mortality (HR = 4.41, 95% CI: 1.35–14.4)
Williams et al. (25)	2017	Retrospective	45 Unspecified subtype	CMR	LGE burden, as determined by CMR-longitudinal strain, is reduced at the apex, compared to basal segments 31.5 ± 19.1% vs. 53.7 ± 22.7%; *p* < 0.001). LGE percentage showed a significant impact on LS (*p* < 0.0001), with a 0.9% decrease in absolute LS for every 10% increase in LGE percentage.
Tavoosi et al. (26)	2020	Retrospective	144	CMR	An abnormal nulling pattern was 100% specific and 40.6% sensitive for cardiac amyloidosis (AUC 0.703, 95% CI: 0.642–0.764), but no significant difference between the subtypes of AL-CA, h-TTR and wt-TTR.
Perugini et al. (27)	2005	Retrospective	25 h-TTR 10 wt-TTR 5 AL 10	Bone tracer scintigraphy	Heart and heart/whole-body tracer retention were significantly higher (*p* < 0.05) in TTR patients as compared with AL patients.
Bokhari et al. (28)	2013	Prospective	45 AL 12 wt-TTR 16 h-TTR 17	Bone tracer scintigraphy	A heart/contralateral ratio ≥ 1.5 was 97% sensitive and 100% specific for cardiac ATTR (AUC 0.992, *P* < 0.0001) in comparison to AL patients.
Castano et al. (29)	2016	Retrospective	229 wt-TTR 72 h-TTR 37 AL 34	Bone tracer scintigraphy	Tc99m PYP imaging was 91% sensitive and 92% specific for cardiac TTR (AUC 0.960, 95% CI: 0.930–0.981) A heart/contralateral ratio ≥1.6 was associated with a poorer prognosis (HR 7.913, 95% CI: 1.679–37.296, *p* = 0.01)
Gilmore et al. (30)	2016	Prospective	1217 wt-TTR 304 h-TTR 258	Bone tracer scintigraphy	Any degree of uptake had a >99% sensitivity for cardiac ATTR (95% CI: 97–100) High grade uptake, when combined with a negative triple test had a specificity of 100% (positive predictive value CI: 99.0–100%)
Scully et al. (31)	2020	Retrospective	100 wt-TTR 34 h-TTR 16 AL 3	Bone tracer scintigraphy	As determined by SPECT/CT, peak SUV > 1.7 had sensitivity of 100% and specificity of 75% for CA [AUC of 0.999 (0.996–1.000)] SUV retention index > 0.14 has a sensitivity of 100% and specificity of 75% for CA [AUC of 0.999 (0.997–1.000)]
Musumeci et al. (32)	2020	Retrospective	55	Bone tracer scintigraphy	CA associated with the Pheo64Leu TTR mutation is detected with a sensitivity of only 10.5% by 99mTc-DPD or 99mTc-HMDP bone scintigraphy
Takasone et al. (33)	2020	Prospective	47 AL 17 wt-TTR 8 h-TTR 22	PET	Positive 11C-PIB uptake on PET, combined with negative corresponding 99mTc-PYP uptake on scintigraphy was seen in all patients with AL-CA Positive 99mTc-PYP uptake, combined with negative corresponding 11C-PIB uptake was seen in all patients with wt-TTR
Antoni et al. (34)	2012	Retrospective	10 AL 7 wt-TTR 1	PET	11C-PIB retention index was significantly higher in amyloidosis, compared with control (mean RI 0.054^−1^ min vs. 0.025 min^−1^, *p* = 0.0007)
Dorbala et al. (35)	2013	Prospective	14 Unspecified subtype	PET	18F-florbetapir retention index was significantly higher in amyloidosis, compared with control (RI median 0.043 min^−1^ vs. 0.023 min^−1^, *p* = 0.0002)
Dietemann et al. (36)	2019	Prospective	12 Unspecified subtype	PET	As assessed using 18F-flutametamol PET, target to background ratio (myocardial/blood pool mean SUV ratio) was significantly higher in CA than control [1.46, interquartile range 1.32–2.06 vs. 1.06, interquartile range 0.72–1.1 (*p* = 0.033)]
Rosengren et al. (37)	2020	Prospective	51	PET	Assessment of SUV retention and retention index of 11C-PIB uptake was 94% sensitive (95% CI: 80–99%) for CA and 93% specific (95% CI: 66–100%) when differentiating from controls. 11C-PIB uptake was significantly higher in AL-CA than in TTR-CA patients (*p* < 0.001)
Lee et al. (38)	2020	Prospective	41	PET	Increased 11C-PIB uptake in AL-CA was associated with poorer clinical outcomes (adjusted HR: 1.185; 95% CI: 1.054–1.332; *p* = 0.005)
Cheyance et al. (39)	2017	Retrospective	45 Unspecified subtype	Cardiac CT	5 min iodine ratio > 0.65 was 100% sensitive and 92% specific for CA. The area under the curve of 5 min iodine ratio for the differential diagnosis of CA from HOCM patients was 0.99 (0.73–1.0; *p* = 0.001)
Treibel et al. (40)	2015	Prospective	53 Unspecified subtype	Cardiac CT	ECV as assessed by cardiac CT was higher in amyloidosis than AS (0.54 ± 0.11 vs. 0.28 ± 0.04, *p* < 0.001) ECV_CT_ tracked clinical markers of cardiac amyloid severity and bone scintigraphy amyloid burden (*p* < 0.001)

*TTE, Transthoracic Echocardiography; CA, Cardiac Amyloidosis; h-TTR, Hereditary Transthyretin; AL-CA, Light Chain Cardiac Amyloidosis; STE, Speckle Tracking Echocardiography; LV, Left Ventricle; EF, Ejection Fraction; GLS, Global Longitudinal Strain; AUC, Area Under Curve; IVS, Interventricular septum; wt-TTR, Wild-type Transthyretin; LGE, Late Gadolinium Enhancement; CMR, Cardiovascular Magnetic Resonance; DPD, 3,3-diphosphono-1,2-propanodicarboxylic acid; LVEF, Left Ventricular Ejection Fraction; LVMI, Left Ventricular Mass Indexed; ECV, Extracellular volume; TDI, Tissue Doppler Imaging; ECVi, ECV at contrast equilibrium; ECVb, ECV at 15-min post-bolus; PYP, pyrophosphate; SPECT, Single-photon emission computed tomography; SUV, Standardized Uptake Value; PET, Positron Emission Tomography; 11C-PIB, (11)C-labeled Pittsburgh Compound-B; HOCM, Hypertrophic Obstructive Cardiomyopathy; CT, Computerized Tomography*.

The aim of this review is to examine the role of each of the imaging modalities utilized in the diagnosis of the two subtypes of CA focusing on the utility of latest techniques available for the diagnosis of amyloidosis.

## Echocardiography

### Transthoracic Echocardiography

**Figure 1:**
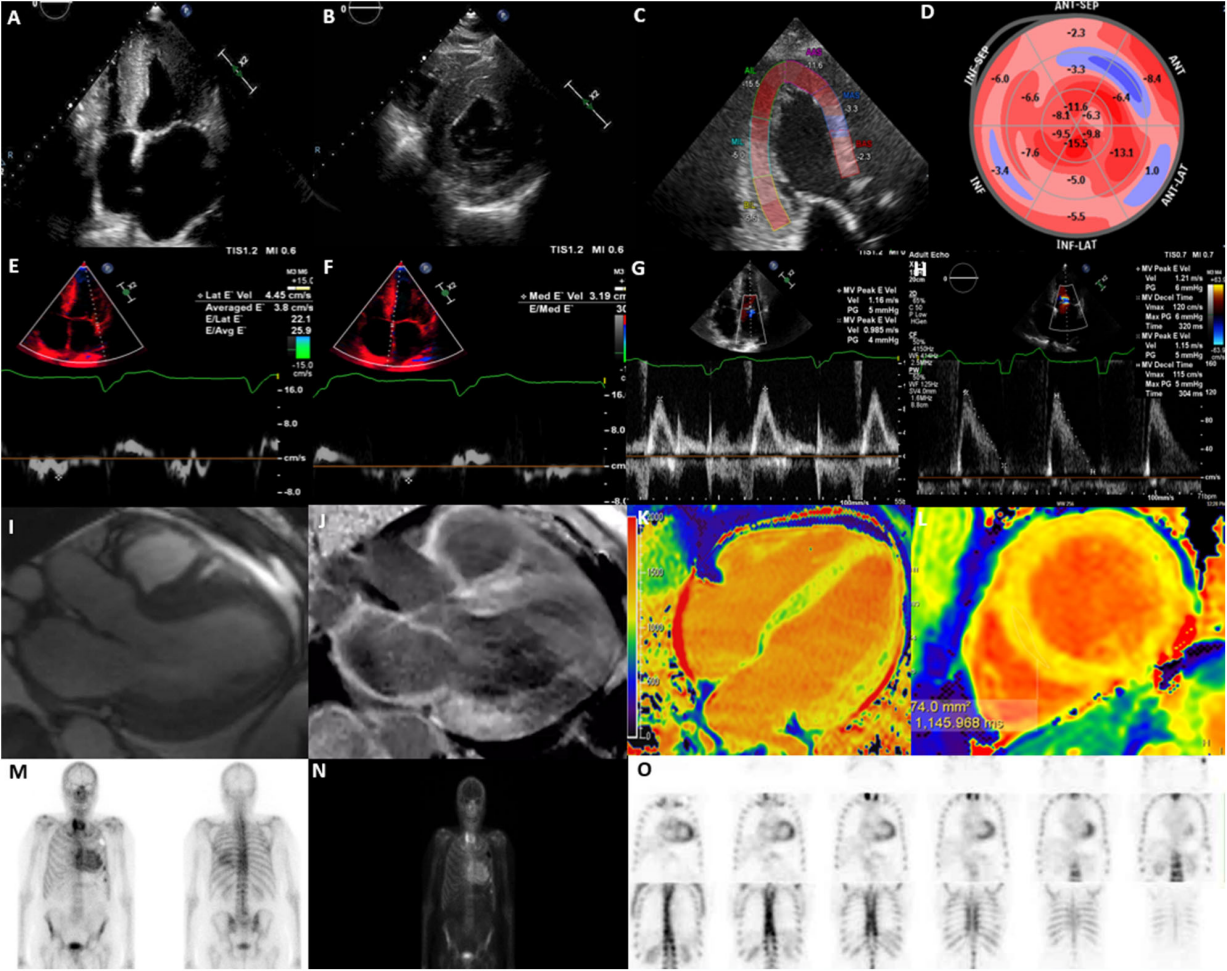
Multi-modality imaging in cardiac amyloidosis. **(A)** Apical 4-chamber view showing increased left ventricular wall thickness (16 mm), dilated left atrium and myocardial speckling. **(B)** Parasternal short-axis view showing increased left ventricular wall thickness. **(C)** Global longitudinal strain on apical 3-chamber view showing presence of apical-sparing. **(D)** Bull's eye longitudinal strain showing evidence of apical-sparing. **(E)** Lateral E' Doppler velocity of 4.45 cm/s (N>10 cm/s). **(F)** Medial E' Doppler velocity of 3.19 cm/s (N>7 cm/s). **(G)** Mitral inflow with an MV peak E velocity of 1.16 m/s (N: 0.6–0.8 m/s). **(H)** Mitral inflow showing a reduction in deceleration time. **(I)** Cine CMR axial 3-chamber view showing concentric increase in left ventricular wall thickness and atrial dilatation. **(J)** Cine CMR axis 3-chamber showing evidence of widespread late gadolinium enhancement, particularly affecting basal inferior, basal anterior left ventricle and left atrium. **(K)** T1 mapping (Modified inversion recovery Look-Locker [MOLLI 3(3)3(3)5] 4-chamber view showing increase in left ventricular native T1 time (region of interest; mid-septum) consistent with amyloidosis; **(L)** T1 mapping MOLLI [3(3)3(3)5)] short-axis view, showing increase in left ventricular mean native T1 time (region of interest; mid septum) consistent with amyloidosis [T1 = 1,145 ms (*N* = 930–1,000 ms)]. **(M)** Bone Tracer Cardiac Scintigraphy using Tc99-DPD showing increased myocardial activity in the ventricles consistent with TTR amyloidosis. **(N)** Whole body 3 h sweep showing increased myocardial activity in the ventricles consistent with TTR amyloidosis; **(O)** Bone Tracer Cardiac Scintigraphy using Tc99-DPD showing increased myocardial activity in the ventricles consistent with TTR amyloidosis. Perugini score is 3 correlating with strong cardiac uptake with mild or absent bone uptake.

TTE is an easily accessible and first line investigative modality that provides an effective two-dimensional measure of cardiac structure and function, routinely is frequently the initial investigative modality for CA. The advantages of TTE include widespread availability, low cost, portability and lack of ionizing radiation, but is limited by patient factors such as body habitus and technical expertise (48). It is useful in the detection of the gross morphological changes seen in this disease such as the echogenic appearance of the myocardium, increased LV wall thickness (IVSd > 12 mm) in the absence of significant LV dilatation and the presence of systolic and/or diastolic dysfunction (49) (Figures 1A,B). The classical pathognomonic TTE finding of “starry and speckled” appearance is historically associated with echogenic proteins that enhance more than the surrounding myocardium in this disease process, but this finding has variable sensitivity and specificity (49). These gross morphological changes are often reflective of more advanced stages of CA.

Measures of LV systolic function such as LV ejection fraction (LVEF), are usually preserved early in the disease process. Typically, impairment of LVEF is usually only apparent in the late-stage of the disease process and is associated with progressive pump failure (41, 49). Indices of diastolic dysfunction may be indicative of early stages of the disease prior to the detection of gross morphological changes but may be non-specific (50) (Figures 1E–G). A higher E/e' and a larger left atrial area, reflective of diastolic dysfunction has been reported in patients with amyloidosis which is attributed to increased stiffness in the myocardium as a result of amyloid protein deposition (7). Additionally, a greater reduction in deceleration time of early filling, and a lower indexed LV mass, have been reported in AL in comparison to TTR amyloidosis, assisting the differentiation of the two pathologies (8) (Figure 1H). Some of these morphological changes, i.e., inter-ventricular septal diameter and measures of diastolic function, i.e., mitral E', E/A ratio and lateral E/e' ratio have been associated with prognosis (11).

### Speckle Tracking Echocardiography

Measures of myocardial deformation indices, i.e., strain and strain rate have been shown to be sensitive in the detection of subclinical impairment of LV systolic function. STE specifically assesses for the LV contraction in the long-axis using global longitudinal strain (GLS), a sensitive measure for the detection of myocardial deformation (51, 52). As such, CA presents with an “apical sparing pattern” that is evident on GLS (Figures 1C,D). Calculations such as the EF:GLS ratio have up to 90% sensitivity and specificity in the diagnosis of CA and has been shown to be the best performance discriminator for CA (AUC, 0.95; 95% CI: 0.89–0.98; *p* < 0.01), independent of disease subtype (9).

In addition to diagnostic utility, GLS can be used as a predictive marker for cardiovascular morbidity and mortality. GLS has been shown to have strong prognostic relevance that can predict all-cause mortality in AL amyloidosis, especially with values less than −14.8% (10). Advanced CA with a GLS<17% have been shown to have a 100% 5-year mortality rate, whereas those with a GLS>17% have a 100% survival rate; further highlighting the short- and long- term prognostic value of GLS in this group (11). These changes in GLS have subsequently been demonstrated to be potentially useful in identification of patients who may be eligibility for hematopoietic stem cell transplant in the AL subgroup (11). Measurements such as relative regional strain ratio, have also been proposed to have prognostic value (12).

There is now emerging evidence highlighting a potential role for myocardial work indices, novel TTE measures of LV systolic function, derived from LV pressure-strain loop analysis, which may be more sensitive than GLS in the diagnosis and prognostication of amyloidosis (13, 14). There is emerging data demonstrating that myocardial external efficiency and total myocardial oxygen consumption are reduced in patients with CA and is associated with abnormal systolic function (13, 14). Hence, despite the limitations of TTE, it is still an invaluable first line investigative tool for the diagnosis and prognostication in those with CA.

## Cardiac Magnetic Resonance Imaging

The utility of CMR is well established in patients with suspected CA and plays an important role in the diagnosis of CA. Despite the limitations of cost, availability and occasional barriers pertaining to non-conditional cardiac devices or severe renal failure, the multi parametric imaging techniques in CMR allow for superior assessment of cardiac structure, function and tissue characterization making it an extremely useful tool in establishing diagnosis (6). The typical CMR findings include globally increased wall thickness, restrictive LV pattern (non-dilated ventricles, preserved LV function, enlarged atria, restrictive filling pattern) global sub-endocardial LGE, atrial septal hypertrophy, increased native T1 mapping values, presence of abnormal myocardial and blood-pool gadolinium kinetics, mild pericardial and pleural effusions (53) (Figure 1I).

### Late Gadolinium Enhancement on CMR

Late Gadolinium Enhancement (LGE) is a CMR finding that occurs due to fibrosis and subsequent expansion of the extracellular space, where the mechanism of LGE in CA is due to infiltration of the amyloid protein (15) (Figures 1J,L). The distribution of delayed enhancement is important as post-ischemic myocardial scar usually involves sub-endocardial or transmural layers, distinct from non-ischemic myocardial diseases that involve global endocardial layers, an important point for differentiating these pathologies (54). A recent systematic review found LGE in any distribution to have a pooled sensitivity of 93% in diagnosing CA, with a reference standard of histology from any organ (55). In amyloidosis, LGE occurs in 3 possible patterns: no LGE, sub-endocardial enhancement, and transmural enhancement, typically occurring along a spectrum. The pattern of LGE may also be useful in differentiating the two subtypes of CA. Fontana et al. demonstrated transmural LGE to be more prevalent in TTR amyloidosis (63% in TTR amyloid vs. 27% in AL amyloid; *p* < 0.0001), as opposed to sub-endocardial LGE which appears to be more prevalent in AL amyloidosis (24% in TTR amyloid vs. 39% in AL amyloid; *p* < 0.05) (15). The Query Amyloid Late Enhancement (QALE) score has been shown to be a powerful prognostic indicator in patients with AL amyloidosis, especially for patients with presence of sub-endocardial LGE pattern, and therefore can guide management (56). Similarly, transmural patterns of LGE have accurately been shown to distinguish TTR from AL amyloid with a high degree of accuracy (*p* < 0.001) (16). Interestingly, Dungu et al. (16) have also shown that right ventricular LGE and atrial LGE is more present in patients with TTR amyloid compared to AL amyloid patients, highlighting the more global effect in TTR amyloid.

Patterns of LGE has also been associated with prognosis with transmural LGE, in AL amyloidosis patients is associated with the poorest prognosis (15, 53). The probability of survival at 24 months in the transmural enhancement group has been shown to be approximately 60% compared with 80% in the sub-endocardial enhancement group, and 90% in the group with no enhancement (15). This may simply reflect the chronicity of the CA disease process- with transmural involvement indicating a longer antecedent disease phase.

CA has also been shown to have a characteristic abnormal pattern of global sub-endocardial late enhancement and abnormal myocardial and blood-pool gadolinium kinetics (53). Patients with CA also have a shorter myocardial nulling time (T1), a longer blood pool T1 and lower post-contrast T1 in comparison to controls, representative of overall myocardial amyloidosis load (22, 53). Normally, myocardium nulls after blood (TI difference >0) in post-contrast LGE images, whereas myocardial nulling before the blood pool (reversed nulling pattern, TI difference <0) is a typical sign of amyloidosis, which has been significantly correlated with higher extracellular volume (ECV) (15, 53). It is often difficult to determine the optimal TI to null myocardium in CA. Conflicting results on morphology and LGE previously reported in the literature were largely explained by misleading findings using the magnitude-only inversion recovery (MAG-IR) LGE technique (15). Nowadays, the widely available LGE technique, phase-sensitive inversion recovery (PSIR), may be helpful in addressing the nulling problems and is the preferred LGE technique to investigate CA, while also providing incremental prognostic information (15).

### T1 and T2 Mapping

Amyloidosis frequently affects the renal system, limiting the use of gadolinium-based agents and assessment of LGE, especially in populations with severe renal impairment (eGFR ≤ 30) (57). Non-enhanced (Native) T1 mapping is a useful quantitative adjunct for the diagnosis of CA, particularly through assessment of degree of fibrosis. As such, Hosch et al. (17) have demonstrated that the myocardium of patients with CA have a significantly increased native T1 relaxation time when compared with control. Additionally, Fontana et al. (18) demonstrated the elevation in T1 is more pronounced in AL amyloidosis when compared with TTR amyloidosis, once again allowing for discrimination of the two subtypes of CA (Figure 1K).

Findings on standard two-dimensional TTE, such as LVEF, LVMI, and E/E' ratio, also correlate strongly with CMR-T1 assessment (19). In addition to this, patients with amyloidosis, without cardiac involvement, also have significantly elevated T1 myocardial relaxation times compared with controls (19). These findings suggest that T1 mapping may have some utility in detecting very early cardiac involvement in patients with a high pre-test probability. Natïve T1 mapping enables differentiation of CA from phenotypically similar Anderson Fabry disease, with the two having diametrically opposite Native T1 times (the fatty infiltration in Anderson-Fabry disease leads to significantly lower T1 time compared to the elevated T1 time seen in CA) as shown by Sado et al. (58).

In addition to T1, native T2 mapping has also been proposed as a potential imaging tool in the diagnosis and characterization of CA. Ridouani et al. (20) demonstrated significantly increased T2 relaxation times in AL, in comparison with TTR amyloidosis, suggestive of a more pronounced myocardial oedema in AL subtype, which may prove a useful non-invasive adjunct to distinguish between the two pathologies. Kotecha et al. (21) found T2 to be predictive of mortality in AL, but not in TTR, even after statistical adjustment for ECV and pro-BNP. Therefore, T2 mapping may have added utility in the diagnosis and prognostication of patients with AL amyloidosis. Other important CMR differentiators for AL amyloidosis from TTR amyloidosis include a higher LVEF, lower LV mass indexed, lower interventricular septal diameter, lower RV free wall thickness and lower bi-atrial areas (16).

One of the main limitations of native mapping (which is more pronounced in T1 compared to T2) is the heterogeneity in reported reference ranges. This is likely due to multiple determinants including specific mapping sequences used, the field strength of the magnet and the inability to distinguish interstitial from myocyte signal intensity (59). This limitation has resulted in recent guidelines recommending each site develop local reference ranges (60). Furthermore, increased myocardial native T1 times, indicative of cardiac fibrosis, may not be specific to amyloidosis since increased myocardial native T1 times have also been detected in early stages of chronic kidney disease. Similarly, the reference range for normal Native T1 in patients with chronic kidney disease is also not well established (60).

### Extracellular Volume

Another measure that potentially facilitates the early detection of CA is the determination of ECV using gadolinium enhanced T1 mapping (both pre-contrast Native T1 and post-contrast T1). ECV is less dependent upon sequence than Native T1 mapping. ECV appears to expand more significantly in amyloidosis, compared to other pathologies; namely diffuse fibrosis processes or non-ischaemic cardiomyopathy (61). One study by Banypersad et al. (23) found significant ECV elevations in patients with confirmed cardiac involvement, compared to healthy controls. Patients with TTR amyloidosis demonstrate a higher ECV in comparison to patients with AL amyloidosis, and this is largely explained by increase cell hypertrophy and hyperplasia in the interstitium (62). ECV also correlated strongly with the presence of LGE (23), and in a recent meta-analysis, was found to have a superior diagnostic performance (51). ECV therefore may be an independent predictor for mortality, (24) and a stronger predictor of outcomes in comparison to LGE and native T1 (63), hence recommended to be routinely assessed in patients with suspected amyloidosis. The limitations in ECV include requiring a haematocrit which should be performed as close in temporal proximity to the CMR acquisition (60).

### CMR Longitudinal Strain

CMR longitudinal strain as assessed by VVI Version 3.0.0 (Siemens) has been shown to appropriately track endocardial and epicardial contours. CMR strain and strain rate have been proposed as sensitive measures to detect early regional myocardial dysfunction in patients with CA. CMR-longitudinal strain has been shown to accurately demonstrate the characteristic apical sparing and base-to-apex gradient, as previously described on TTE (25). Furthermore, CMR strain analysis can assist with diagnosis of LGE-positive CA with measures such as peak circumferential strain described as more sensitive that LGE assessment in early stages of the disease (64). Additionally, basal segment strain parameters have also been shown to accurately identify presence of cardiac involvement in patients with systemic amyloidosis (65).

## Nuclear Imaging

### Bone Tracer Cardiac Scintigraphy

Tc99-PYP scanning is a sensitive imaging modality for identifying presence of TTR subtype of CA. It is performed with various technetium-99m-labeled phosphonates; including 99mTc- 3,3-diphosphono-1,2-propanodicarboxylic acid (DPD), 99mTc-PYP, and 99mTc-labeled HDMP. TTR amyloidosis has been shown to have avidity for these radiotracers, whilst AL amyloidosis has at most minimal avidity (27). The semi-quantitative Perugini visual scoring system (2005) is widely used and compares the degree of cardiac uptake with bone uptake; where Score 0 correlates with absent cardiac uptake with normal bone uptake; Score 1 correlates with mild cardiac uptake which is inferior to bone uptake; Score 2 correlates with moderate cardiac uptake with attenuated bone uptake and Score 3 identifies strong cardiac uptake with mild or absent bone uptake (27, 66). A quantitative assessment can also be performed by calculating the heart to contra lateral (H/CL) ratio, using a region of interest over the heart, and another over the contralateral hemi-thorax (67). Using this ratio, Bokhari et al. (28) have determined that a H/CL ratio>1.5 was 97% sensitive and 100% specific for TTR amyloidosis. Furthermore, a H/CL ≥ 1.6 has also been shown to be associated with poorer prognosis amongst those with TTR (29). Therefore, similar to other cardiac imaging modalities, bone-tracer cardiac scintigraphy metrics provide both diagnostic and prognostic information in patients with CA.

Gillmore et al. (30) described >99% sensitivity for TTR amyloidosis with any degree of cardiac uptake, however specificity was reduced to 68% due to a low level of uptake in some patients with AL amyloidosis. The specificity of diagnosing TTR amyloidosis increases to 97% with only high-grade uptake (Perugini Score 2–3). Furthermore, the addition of high-grade uptake with a negative “triple test” (serum and urine immune-fixation, and serum free light chain assay), results in a specificity of 100%, potentially mitigating biopsy requirement in such populations. Similar to the typical “apical sparing” seen on TTE, TTR characteristically has increased radionuclide uptake in the mid and basal segments when compared to apical segments (68, 69).

Single-photon emission computed tomography (SPECT)/CT assessment may offer improved diagnostic accuracy in addition to the conventional planar images on nuclear scans. A retrospective study assessing SPECT/CT quantification of DPD-scintigraphy found that the peak standardized uptake value (SUV) increased from Perugini grade 0–2 (*p* < 0.001), thereby increasing the diagnostic accuracy. Furthermore, the SUV retention index calculated on the peak SUVs of cardiac tissue, vertebra and para-spinal muscle, was found to increase across all grades. Cut-offs of peak SUV > 1.7 and SUV retention index > 0.14 detected presence of CA with a sensitivity of 100% and specificity of 75%, superior to conventional planar quantification where a H/CL ratio > 0.97 was described to have only a 100% sensitivity but only a 38% specificity (31). Therefore, the implementation of SPECT/CT to DPD scanning adds accuracy to the diagnostic process of CA.

While bone scintigraphy appears to be an overall effective method for identifying TTR amyloidosis, there is emerging evidence that this may not apply to all variants. CA associated with the “Pheo64Leu TTR” mutation, for example, was detected with a sensitivity of only 10.5%, with majority of the cohort having absent or low uptake (32). These findings highlight the emerging role of SPECT/CT in the diagnostic process for the majority of subtypes of CA.

### Positron Emission Tomography

Cardiac PET scans involve a nuclear technique that measures physiological blood flow and metabolism of myocardial tissue and has been shown to have diagnostic value in CA (36) (Figures 1M–O). Several pilot studies have found PET imaging to be effective as a tool in both diagnosing CA, and useful in differentiating between TTR and AL amyloidosis (35–37). Whilst Fludeoxyglucose (FDG)-PET scans have been shown to have high organ uptake in patients with AL amyloidosis, they carry overall low sensitivity and specificity rates, as they have low differentiation between physiological and pathological uptake (70). Despite the low availability in most centers, increased uptake of 11-C-Pittsburgh compound B (11c-PiB), 18F-florbetapir, 18F-florbetaben and 18F-flutemetamol via PET have been shown in patients with CA compared to control populations (34–36, 71). A recent meta-analysis of six studies with 98 patients demonstrated a high pooled sensitivity of 95% and specificity of 98% (72). Furthermore, Rosengren et al. (37) demonstrated 11cPiB PET to carry 100% diagnostic accuracy for AL amyloidosis (95% CI: 88–100%), which was also significantly higher when compared to the TTR population (*p* < 0.001). In addition, Lee et al. (38) found increasing 11c-PiB uptake to be associated with poorer clinical outcomes (adjusted HR: 1.185; 95% CI: 1.054–1.332; *p* = 0.005**)**.

## Cardiac Computed Tomography

There is currently limited evidence for the role of cardiac computed tomography (CCT) in the diagnosis of CA, despite its wide utility in combination with nuclear imaging. The majority of evidence lies with myocardial iodine ratios, which involve the assessment of myocardial iodine concentration (MIC) approximately 5 min after peripheral iodine administration, done on pre-contrast, arterial and 5 min dual-energy computed tomography acquisition. As several studies have demonstrated a link between myocardial attenuation and iodine administration in patients with CA, MIC thereby provides a quantitative measure of total myocardial involvement (73). MIC has been shown to accurately distinguish CA from other subtypes of cardiomyopathies with a high sensitivity rate of 100% and specificity of 92% (39). Second to this, myocardial ECV as assessed by CCT has been shown to accurately correlate with amyloid burden as assessed by bone scintigraphy (40). Further studies in CCT are required to appropriately demonstrate its effectiveness and utility in the diagnostic process.

### Dual Aortic Stenosis and Cardiac Amyloid Pathology

In addition to appropriate diagnosis of CA, it is also imperative to identify other disease processes, such as aortic stenosis (AS), through non-invasive imaging modalities. Calcific AS, similar to CA, manifests both clinically and phenotypically as a disease of the elderly population. A meta-analysis of 609 patients by Ricci et al. (74) showed that the presence of dual AS-CA had a 9% prevalence, consisting of 100% TTR amyloidosis patients. The presence of dual AS-CA pathology has additionally been associated with a poorer prognosis, in comparison to CA alone (median survival 22 vs. 53 months) and therefore should be considered in all cases (75, 76). Baseline TTE parameters suggestive of CA, distinct from AS include an elevated RA diameter, lower AV pressure gradient, higher tricuspid regurgitant grade, lower stroke volume index and higher LV mass indexed (77). Some of the important CMR findings include stroke volume index and ECV, which have both been shown to reliably differentiate AS from CA-AS, with a discriminative power of 0.773 and 0.756 AUC, respectively (77). Other CMR findings that are more suggestive of CA include severe LV increased wall thickness, the presence of global transmural LGE, elevated native myocardial T1, ECV > 50%, and a greater reduction in myocardial contraction fraction (76). On cardiac CT, a higher myocardial ECV has been shown to reliably identify the presence of AS-CA, outperforming conventional TTE, as shown by Scully et al. (78). Similarly, on DPD bone scintigraphy, occult wt-TTR CA has been shown to have a prevalence of 6% in patients with calcific-AS (79). These findings highlight the importance of identification and differentiation of these two pathologies, as it may impact on clinical and surgical management options.

## Conclusion

The diagnosis of CA is often challenging, but there is a large body of emerging evidence for the role of multimodality imaging in achieving the diagnosis with higher sensitivity and specificity. The development of novel techniques with unique qualities have now even allowed for the differentiation between CA from other cardiomyopathies, identification of the different subtypes of CA and prognostication in these patients. The role of multi-modality imaging for the diagnosis of CA is also growing, but further studies in this field are clearly required to better delineate the optimal investigative tools for the diagnosis and differentiation of CA.

## Author Contributions

SK and IW: drafting of manuscript. AB, HC, and FP: revisions of manuscript. GG: supervision. TT: supervision and revisions of manuscript. All authors contributed to the article and approved the submitted version.

## Conflict of Interest

The authors declare that the research was conducted in the absence of any commercial or financial relationships that could be construed as a potential conflict of interest.
